# A Century of Technical Publishing

**Published:** 1920-12-25

**Authors:** 


### A CENTURY OF TECHNICAL PUBLISHING.

The Christmas publishing season coincides with the issue of an interesting volume describing a century of book production by Messrs. Charles Griffin & Co., whose firm has been mainly occupied with technical and scientific books. A number of well-known authorities in pure and applied science contribute to the volume, but our concern is with the concluding chapter, wherein a member of the Royal College of Physicians describes the work done during the nineteenth century by the firm in the department of medical and sanitary science.

Before the firm began publishing, they combined bookselling with the sale of chemicals, and their premises in Glasgow included a chemical museum and a lending library. The early publications were mainly treatises on chemistry, but medical books began to be issued in 1845. In 1858 Surgeon-General Longmore published his "Sanitary Contrasts of the European War." It is interesting to recall that the firm's "Encyclopædia Metropolitana" included an article on "The Elementary Principles of Medicine," by Dr. Robert Williams, of St. Thomas's Hospital, which is said to have been the same from which Dr. William Aitken's "Science and Practice of Medicine" originated. The Indian Mutiny led to a demand for books from the Army Medical School, and Griffin's issued "The Surgeon's Pocket-book," by Major T. H. Porter, which was in use up to the time of the Boer War. A pocket series of medical reference books quickly followed, including works on medical jurisprudence, toxicology, and tropical practice. These were followed by a series on public health and hygiene, of which Blyth's "Dictionary of Hygiene and Public Health" was perhaps the chief and most authoritative. The medical publications now increased in number, and included the "Quarterly Journal of Anatomy and Physiology," which Sir William Turner, and then Professor Alexander Macalister, of Cambridge, edited. Sir Dyce Duckworth's "Treatise on Gout" and Sir Victor Horsley's "Structure and Functions of the Brain and Spinal Cord" were also published by the firm. Dr. Charles Mercier, a regular contributor to The Hospital, issued his "Lunatic Asylums: their Organisation and Management" through Griffin's, which also published the work of his old friend Sir Bryan Donkin on "Diseases of Childhood."

We must rather pass to the nursing section, which began with Dr. Laurence Humphry's "Manual of Nursing, Medical and Surgical." Certain other volumes mark the recognition of new subjects round which separate literatures continue to grow, such as Scott Riddell's "Manual of Ambulance" and "How to Become a Woman Doctor," for which Dr. W. J. Fenton wrote the preface. Household medicine and books for wives and mothers, together with volumes on infancy and for health visitors, indicate the demands of the present century for sound information in popular form and for guides to the new professions for women to which modern ideas of preventive medicine have led.

